# Uveal Versus Cutaneous Melanoma; Same Origin, Very Distinct Tumor Types

**DOI:** 10.3390/cancers11060845

**Published:** 2019-06-19

**Authors:** Monique K. van der Kooij, Frank M. Speetjens, Sjoerd H. van der Burg, Ellen Kapiteijn

**Affiliations:** 1 Department of Medical Oncology, Oncode Institute, Leiden University Medical Center, Albinusdreef 2, P.O. Box 9600, 2300 RC Leiden, The Netherlands; s.h.van_der_burg@lumc.nl; 2 Department of Medical Oncology, Leiden University Medical Center, Albinusdreef 2, P.O. Box 9600, 2300 RC Leiden, The Netherlands; f.m.speetjens@lumc.nl (F.M.S.); h.w.kapiteijn@lumc.nl (E.K.)

**Keywords:** uveal melanoma, metastatic uveal melanoma, cutaneous melanoma, driver mutations, liver metastases, adaptive immunity

## Abstract

Here, we critically evaluated the knowledge on cutaneous melanoma (CM) and uveal melanoma (UM). Both cancer types derive from melanocytes that share the same embryonic origin and display the same cellular function. Despite their common origin, both CM and UM display extreme differences in their genetic alterations and biological behavior. We discuss the differences in genetic alterations, metastatic routes, tumor biology, and tumor-host interactions in the context of their clinical responses to targeted- and immunotherapy.

## 1. Melanocytes and Their Cellular Function

Melanocytes originate from neural crest cells and are present in various parts of the human body, including the skin, eyes, cochlea, mesencephalon, and the heart. There they are responsible for the synthesis of melanin pigments within organelles called melanosomes. In the epidermis, melanocytes transfer these melanin-containing melanosomes to neighboring keratinocytes. This ensures homogeneous pigmentation, determines skin color and protects against the harmful effects of ultraviolet radiation (UVR) [1]. In the eye, melanocytes are found in the conjunctiva and all areas of the uvea (the iris, ciliary body, and choroid). Conjunctival melanoma is distinct from uveal melanoma (UM) and shares more commonalities with cutaneous melanoma (CM) [2].

The quantity and quality of melanin pigment in the iris determines its color. In contrast to the skin, the iris color is not influenced by sun exposure. The variance in melanin expressing uveal melanocytes is associated with the occurrence of various ocular diseases, including age-related macular degeneration and uveal melanoma [3,4]. Both CM and UM arise from melanocyte transformation and represent deadly forms of cancer. 

## 2. Genetic Alterations and Treatment Implications

CM and conjunctival melanoma are genetically distinct from UM. The majority of CM cases harbor mutations in proteins associated with the mitogen-activated protein kinase (MAPK) pathway. This is an important intracellular signaling pathway involved in cell growth, differentiation, and survival. Oncogenic activation of the MAPK pathway may occur via multiple mechanisms but most commonly is driven by a constitutively activated mutated *BRAF* kinase. *BRAF* kinase mutations are present in 40–60% of the CM patients, 97% of which is located in codon 600.

**Figure 1:**
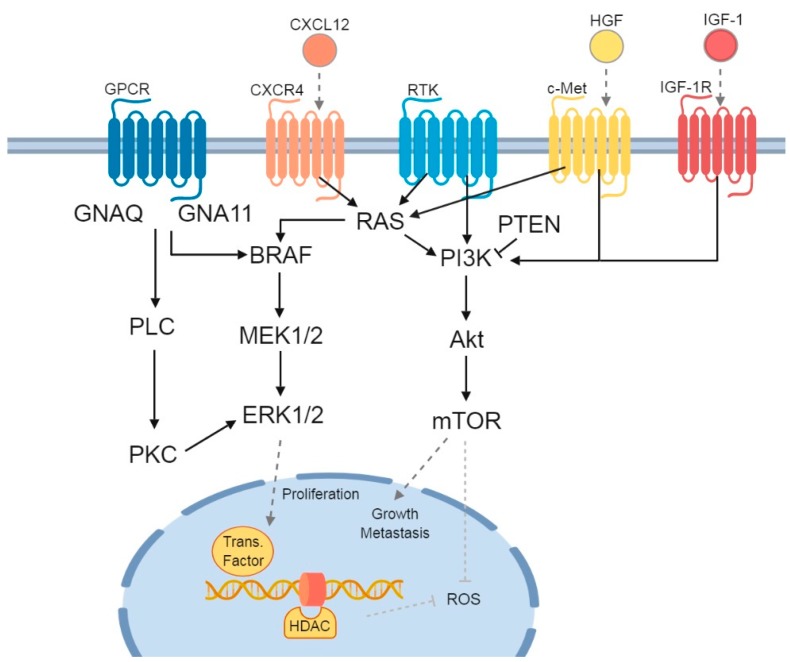
Signaling pathways and receptors involved in uveal melanoma (UM) and cutaneous melanoma (CM). Three main signaling pathways affected in UM and/or CM patients are depicted. G protein-coupled receptor (GPCR) with its Guanine nucleotide-binding proteins: the first is the Guanine nucleotide binding protein (*GNAQ*) and subunit alpha-11 (*GNA11*), which downstream activate Phospholipase C (PLC) and Protein Kinase C (PKC). The second is the mitogen-activated protein kinase (MAPK) signaling pathway, consisting of *BRAF-MEK1/2-ERK1/2*. Finally, there is the *PI3K/Akt/mTOR* pathway, which can be influenced by both RAS (from the MAPK signaling pathway) and phosphatase and tensin homolog (PTEN). The previously described chemokine receptors and their influence on the signaling pathways are added: C-X-C chemokine receptor 4 (CXCR4), with its C-X-C Motif Chemokine Ligand 12 (CXCL12), tyrosine-protein kinase Met (c-Met) and its ligand Hepatocyte Growth Factor (HGF), and Insulin-like Growth Factor-1 Receptor (IGF-1R), with Insulin-like Growth Factor-1 (IGF-1). In the nucleus, the ERK1/2 stimulates transcription factors, while both histone deacetylase (HDAC) and mechanistic target of rapamycin (mTOR) inhibit the formation of Reactive Oxygen Species (ROS). Figure was created with BioRender.com.

*BRAF*-mutated melanoma tends to exhibit distinctive clinical features and is characterized by a more aggressive biological behavior than *BRAF* wild-type (WT) melanoma. *BRAF*-mutated melanoma may be associated with shorter overall survival and adverse prognostic factors, but this is still under investigation [5,6,7,8]. The second most common MAPK pathway aberration in CM is mutated *NRAS*, occurring in 15–30% of patients (Figure 1) [9,10,11,12]. Melanoma with mutations in the stem cell factor receptor tyrosine kinase gene (*KIT*) represents a relatively rare subset, seen in roughly 20% of mucosal, acral, and chronically sun-damaged skin [13].

The discovery that many CM are caused by a mutation in *BRAF* kinase has led to the development of selective inhibitors of the *BRAF* V600-mutated kinase (vemurafenib, dabrafenib, and encorafenib) and inhibitors of the downstream *MEK* kinase (trametinib, cobimetinib, and binimetinib). BRAF inhibition results in high response rates in patients with a *BRAF* V600E or V600K mutation; however, most patients ultimately develop acquired resistance. The combination of BRAF and MEK inhibitors is more effective in forestalling the development of acquired resistance when compared to BRAF monotherapy [14]. Five large phase III randomized controlled trials reported a median progression free survival for the combination treatment with BRAF and MEK inhibition of 9.3–11.4 months whereas this was 5.8–8.8 months for treatment with a BRAF inhibitor and placebo [15,16,17,18,19]. The treatment with KIT inhibitors improved the overall survival of patients with *KIT*-mutated gastro-intestinal stromal tumors. Following this success, multiple trials have shown that patients with metastatic melanoma harboring a *KIT* mutation were responsive to therapy with KIT inhibitors imatinib, sunitinib, dasatinib, and nilotinib [13]. The response rates in patients with metastatic melanoma are around 20–25%, when all *KIT* genetic lesions are considered, and reach 35–50% in melanomas with a *KIT* mutation in exon 11 or 13 [20,21,22,23,24].

Mutations in *BRAF* V600E occur in 29–50% and mutations in *NRAS* occur in up to 18% of the patients with a conjunctival melanoma. *KIT* mutations have only been reported in one conjunctival tumor [25,26]. As it is a rare form of ocular melanoma, clinical data after BRAF inhibition is scarce. Two case reports show mixed results [27,28]. However, the genetic similarities suggest that treatment regimens used for metastatic CM should be further investigated in metastatic conjunctival melanoma.

In UM, the most commonly mutated genes are *GNA11*, *GNAQ*, *BAP1*, *EIF1AX*, and *SF3B1*. More than 90% of the UM exhibit a mutation in *GNA11* or *GNAQ*, which activate signaling between G-protein-coupled receptors and downstream effectors as well as upregulate signaling of the MAPK pathway (Figure 1) [29,30]. These mutations occur mutually exclusive in the majority of uveal melanomas, and are considered an early event in the development of UM. Mutations in *GNAQ* and *GNA11* are not associated with a worse prognosis or with the development of metastatic disease [31,32,33,34].

However, primary UM can be stratified into four distinct, clinically relevant molecular subtypes with a significant difference in metastatic rate and prognosis [30]. Class 1A and 1B tumors retain a differentiated melanocyte phenotype, with a disomy of chromosome 3. They are further distinguished by alterations in either *EIF1AX* or *SF3B1*, respectively, with 1A having a lower metastatic rate when compared to 1B. Class 2 UM is associated with a high metastatic risk and is characterized by a monosomy of chromosome 3, followed by aberrancies in *BAP1* expression and global DNA methylation. A further subdivision can be made into class 2A and 2B based on chromosome 8q copy number alterations, RNA expression, and cellular pathway activity profiles [35]. With Class 2B having a higher metastatic rate when compared to Class 2A [35,36,37].

As most UM are characterized by mutations in *GNAQ* or *GNA11*, therapies that target downstream effectors of these pathways such as *MEK*, *Akt*, and protein kinase C (PKC) are being investigated. Unfortunately, the results have been disappointing with response rates generally less than 10% [38,39]. A promising new target in UM could be epigenetic dysregulation. As previously mentioned, somatic mutations in the tumor suppressor gene *BAP1* are correlated with metastatic behavior [40]. The loss of *BAP1* seems to sensitize UM cell lines to treatment with histone deacetylase (HDAC) inhibitors. HDAC induces a G1 cell cycle arrest with an increased cyclin D1, impaired cell proliferation, growth reduction, and induction of apoptosis in UM both in vivo and in vitro [41,42,43].

Treatment with HDAC inhibitors might prove to be beneficial for both UM and CM, as the balance between histone acetylation and deacetylation is altered in multiple cancer types. This balance defines the level of acetylation of histone and therefore plays a critical role in the regulation of gene expression [44]. While histone acetyltransferases (HAT) mediated acetylation is associated with gene transcription, HDAC-mediated histone deacetylation is associated with gene silencing. Inhibition of HDAC was shown to block tumor cell proliferation and differentiation. Currently, there are four HDAC inhibitors approved by the FDA for treatment of cancer; vorinostat, romidepsin, belinostat for T-cell lymphoma, and panobinostat for multiple myeloma [45]. Currently, several trials are studying the effect of HDAC inhibition in patients with UM or CM. Furthermore, there is pre-clinical evidence that combining HDAC inhibitors with conventional immunotherapies, targeted therapies, or cyclin-dependent kinase (CDK) inhibitors might work synergistically [46,47,48].

## 3. Biological Parameters Underlying Metastasis

Cutaneous and ocular melanomas have distinctly different clinical courses. For both CM and UM, the development of metastatic disease is an important determinant of the clinical course and survival. CM tends to spread via the lymphatic system, mostly to the lungs, brain, lymph nodes, and soft tissue, with 14–20% of patients developing liver metastases [49]. Because there are no lymphatics in the uveal tract, ocular melanoma spreads hematogenously, resulting in the liver as the predominant metastatic site (89% of cases) [50].

The striking liver tropism of UM metastasis is currently not fully understood. In 1889, Paget introduced the concept of ‘’seed and soil’’, which proposed that the spread of tumor cells is governed by interaction and cooperation between the tumor and the host organ [51]. More recent studies have provided a better understanding of the process of metastatic spread of multiple cancer types, including melanoma [52]. One of these studies showed that some tumors succeed in creating a premetastatic niche in the liver. They manipulate the microenvironment of different organs to render them more permissive to metastatic outgrowth before the cancer cells actually enter the organ. It was shown that integrin expression profiles of circulating plasma exosomes isolated from amongst other CM and UM could be used as a prognostic factor to predict sites of future metastasis [53].

Furthermore, a wide variety of tumors express chemokine receptors corresponding with the expression of their respective ligands in the organs bearing the highest frequency of metastases. Chemokine receptors might also influence the overall survival in patients, and may present as potential targets for treatment.

(1) CCR4-CCL17/CCL22 axis: in CM, it was shown that CCR4 overexpression might enhance the tumor’s potential to metastasize to the brain [54].

In UM, no correlation between this axis and metastatic pattern has thus far been described [55].

(2) CCR7-CCL19 axis: in CM, the CCR7-CCL19/CCL21 axis is associated with regional lymph node metastases [56,57].

In UM, the expression of CCR7 seemed to be correlated with the development of liver metastases. Both in CM and UM, this axis has been correlated with a worse patient outcome [58,59].

(3) CCR10-CCL27 axis: in a CM preclinical model, it was shown that CCR10 might play an important role in sustaining tumor viability, in protecting cells from the immune response, and in the dissemination to the draining lymph node. High expression of CCR10 was associated with a worse overall survival [57,60,61].

In UM, no correlation was found between the presence of CCR10 and/or CCL27 and the formation of liver metastases [62].

(4) CXCR3-CXCL9/CXCL10 axis: stimulation of this axis has been described to be have both pro-tumor and anti-tumor effects. This may be due to the different effects of the ligands on CXCR3. CXCL9 predominantly mediates lymphocytic infiltration and suppresses tumor growth. The induction of both CXCL9 and CCL10 expression was also seen in CM patients that responded well to interleukin 12 immunotherapy [63]. Furthermore, stage III CM patients with CXCL10 expressing CD8 T cells had a better overall survival. Conversely, CXCR3, the receptor for both CXCL9 and CXCL10, is associated with thicker primary tumors, the absence of lymphocytic infiltration, and the presence of distant metastases. It has been shown that the anti-tumor effect of this axis is induced by paracrine activation by immune cells, while the pro-tumor effect is caused by autocrine signaling mainly through the CXCR3A ligand in cancer cells [64]. The selective targeting of CXCR3A was therefore suggested to be an effective treatment option in metastatic disease.

In UM, it has been shown that CXCL10 is upregulated in a T-cell-rich environment. Recently, it was shown that in UM, mainly activated macrophages express this lymphocyte-homing chemokine CXCL10. Furthermore, CXCL10 expression may serve as an independent risk factor, inversely correlated with survival [36].

(5) CXCR4/CXC7-CXCL12 axis: in CM, high CXCR4 expression is associated with the presence of tumor ulceration, thicker lesions, as well as shorter disease-free survival, time to metastasis, and overall survival. Furthermore, its expression is associated with the development of liver and lung metastases [65,66].

The expression of CXCR4 on UM cells and the presence of CXCL12 in the liver offers an explanation for the selective colonization of the liver by UM. Interactions between CXCR4 and CXCL12 stimulate tumor cell migration and invasion of basement membrane preparation by increasing the formation of cell adhesion molecules like matrix metalloproteinases [59,67]. CXCL12 also stimulates proliferation and survival of CXCR4 positive tumor cells [68,69,70]. Furthermore, chemotaxis of uveal melanoma cells could be inhibited by anti-CXCR4 [59].

(6) c-Met, a receptor for hepatocyte growth factor (HGF): In CM overexpression of c-Met is associated with tumor growth and metastasis. Inhibition of HGF induced c-Met proliferation reduced melanoma cell line migration and invasion in vitro [71].

In UM c-Met also promotes tumor invasion and stimulates tumor growth [72]. The expression of c-Met in primary UM increases the risk of subsequent liver metastasis [73]. Cabozantinib is a tyrosine kinase inhibitor that targets the MET, AXL, and vascular endothelial growth factor (VEGF) receptors. In CM cells it inhibits HGF-induced migration and invasion [74], while in an UM xenograft model, it was shown to reduce hepatic metastasis [75]. A recent phase II randomized discontinuation trial in which the MET/VEGF receptor inhibitor cabozantinib was tested, revealed clinical activity in both metastatic CM and UM patients [76].

(7) Insulin-like growth factor-1 (IGF-1) plays an important role in tissue growth, and increases the risk for the development of many tumor types, including CM [77]. Both in CM and UM the serum IGF-1 level functioned as a potential predictive biomarker for metastatic disease. Strikingly, whereas metastatic UM patients displayed lower IGF-1 serum levels when compared to healthy controls, the IGF-1 serum levels were higher in metastatic CM patients [78,79]. In UM, a high expression of the IGF-1 receptor (IGF-1R) was found in hepatic metastasis and related to death due to metastatic disease [80,81,82]. The IGF/IGF-1R axis has been a target for new treatment combinations in both CM and UM. In CM, IGF-targeting agents have been used in combination with other treatment modalities, as it plays a role in both primary and acquired treatment resistance [83]. Preclinical research shows promising results whenIGF-1R inhibition is combined either with PI3K inhibition, Stat3 blocking, or chemotherapy (temozolomide) [84,85,86]. In metastatic UM, a trial treating patients with an anti-IGF-1R antibody (IMC-A12, cixutumumab), was conducted. However, the final results have not yet been published (NCT01413191).

Hypoxia-inducible factor (HIF) plays a key role in tumorigenesis and metastasis in multiple types of cancer [87]. It plays an important role in the development of CM from melanocytes. Even at normal oxygen levels, HIF activity is increased in melanoma, thereby accelerating the invasion of tumor cells into adjacent tissues and providing sufficient blood supply [88,89]. Recently, FBXO22 was introduced as a possible new treatment option for CM as it is supposed to regulate the expression of HIF [88].

In UM it was shown that relative activity of hypoxia differentiated the subgroups, irrespective of chromosome 3 status [35]. Both the previously mentioned c-Met and CXCR4 are important surface mediators of hypoxia-induced migration, invasion, and metastasis [90,91]. In addition, elevated mRNA expression of both MET and CXCR4 was found in patients with a poor prognosis and the expression levels of CXCR4, c-Met, and HIF-1 were higher in the primary tumor of patients with a subsequent metastasis. Furthermore, in cell cultures hypoxia can induce c-Met and CXCR4 expression, while these effects were inhibited by a HIF pathway inhibitor (arylsulfonamide 64B) both in vitro and in an in vivo orthotopic mouse model. In vivo treatment resulted in inhibition of primary UM growth, less liver metastasis formation, and a better survival [92].

## 4. The Impact of the Immune System

### 4.1. Primary Tumor

The distribution of immune cells varies between different tumor types. In CM, the role of the adaptive immune response in controlling tumor progression has gained a lot of attention over the past decades. In primary CM the presence of CD3+CD8+ lymphocytes, specifically activated (HLA-DR expressing) CD8+ T cells, in both the tumor and the stroma was correlated with disease-specific survival [93].

Multiple studies have investigated the role of immunosuppressive regulatory T cells (Treg) in primary CM, with conflicting results. This might be due to differences in phenotypic markers used or technical differences in staining and analyzing, as the two papers showing no difference identified Tregs as FoxP3+ cells and the paper showing a difference identified these cells as being CD25+FoxP3+ [94,95,96]. This emphasizes the need for a robust gating strategy for the analysis of Tregs [97].

Additionally, the role of macrophages has been investigated. There are two major subtypes of macrophages, being the macrophages that support an effective antitumor response (M1) and the macrophages that promote tumor growth (M2). In the early development of CM, the M1-recruited macrophages shift to the M2 phenotype, thus favoring tumor proliferation and dissemination [98].

In contrast to CM, the pronounced infiltration of UM by immune cells is associated with a poor prognosis [99]. Primary UM with monosomy 3 is associated with infiltration with a variety of immune cells, including CD8+, CD4+, and CD3+CD8-FoxP3+ T cells as well as CD68+CD163+ M2 macrophages. The Class 2B tumors that display a gain in the copy number of chromosome 8q are associated with the increased expression of macrophage-attracting chemokines and a stronger influx of myeloid cells, whereas additional aberrations in BAP1 expression seem to drive T cell infiltration, irrespective of the chromosome 3 status [100]. The presence of a CD3+ immune infiltrate in Class 2 tumors, while nearly absent in Class 1 tumors, coincides with the increased gene expression of human leukocyte antigen (HLA), suggesting the local production of type II interferon [101]. Notably, the infiltration with all these immune cells is collectively increased, the balance of the different cells was of no clinical relevance [102,103], although one study suggested that the presence of the immunosuppressive Tregs within a subgroup of COX2+ primary UM forms an independent prognostic factor for worse overall survival [104].

### 4.2. Metastatic Melanoma

In many metastasized tumors, including CM, the presence of effector T lymphocytes is beneficial, including CD8+ T cells and CD4+ helper T cells. The presence of CD4+CD25+ Tregs may be detrimental [105]. Our group recently identified four intratumoral parameter profile that was associated with a better survival in metastatic CM patients. Namely, the presence of tumor infiltrating CD3+CD8+FoxP3− T cells, galectin-9+ dendritic cells (DC)/DC-like macrophages, a high CD14+CD163− (M1)/CD14+CD163+ (M2) macrophage ratio, and the expression of galectin-3 by tumor cells. Patients with three or four of the described parameters present displayed the longest overall survival [106].

Currently, one of the most established treatments for metastatic CM is via immune stimulation with checkpoint blockers. This type of treatment relies on antigen-specific T cell responses by alleviating tumor-induced immunoregulatory mechanisms [107]. Immune checkpoint blockade can achieve durable responses in many CM patients and has shown to improve overall survival in this patient group. The first blocking antibody that was tested and approved for the treatment of cancer patients was against cytotoxic T-lymphocyte antigen-4 (CTLA-4). CTLA-4 increases the activation threshold of T cells, reducing immune responses to weak antigens such as self- and tumor antigens. The second blocking antibody introduced into the clinic was targeting Programmed death 1 (PD-1). While CTLA-4 mainly plays a role in the activation phase in the draining lymph node, PD-1 predominantly regulates the effector phase of T cell responses within peripheral tissues. PD-1 binding with its ligands decreases the magnitude of the immune response in T cells that are already engaged in an effector T cell response. This results in a more restricted T cell activation compared to CTLA-4 blockade, which can lead to an unspecific activation of T cells in the lymphoid organs. This could explain why PD-1 inhibition shows fever side effects and greater antitumor activity than CTLA-4 inhibition [108,109,110,111]. The updated survival data from the CheckMate 067 study showed a 3-year overall survival of 58% in the patients treated with anti-PD1 and anti-CTLA4, 52% in patients with anti-PD1 monotherapy and 34% in patients treated with anti-CTLA4 monotherapy [108].

Treatment with these checkpoint blockers has been investigated in UM. Unfortunately, the clinical response rates reported for anti-PD1 or anti-CTLA4 are unimpressive, with no significant OS benefit in UM patients [112,113,114,115,116,117,118,119,120]. A trial investigating the combination of these checkpoint inhibitors is still ongoing (NCT01585194).

Little is known about the immune microenvironment of metastatic UM (mUM). Therefore, reasons underlying the poor response to immunotherapy are unclear and have led to speculation that UM may represent an immunotherapy resistant form of melanoma. Several recent findings might help to shed some light on why UM does not respond to immunotherapy like CM.

High mutational burden is predictive of the response to immune checkpoint inhibitors across multiple cancer types [121]. The neoantigens that derive from these tumor-specific mutations are potential targets for anti-tumor immune responses, as they are foreign to the immune system. Cutaneous melanoma is one of the tumors with the highest somatic mutation prevalence [122]. In contrast UM lacks the UV-radiation mutation signature and has a low mean somatic mutation rate [123]. The lack of these targets could be a possible explanation as to why immune stimulation with checkpoint inhibitors alone is not sufficient in UM, while it can be sufficient in CM. However, low-mutational burden may also lead to the spontaneous activation of neoantigen-specific T cells [124,125].

In a recent pilot study, the immune profile of both CM and UM metastases was characterized. Overall, it seemed that the CD8 infiltration in both tumors was similar. Interestingly, the PD-1 expression levels were lower in mUM patients than those observed in metastatic CM (mCM). Furthermore, it also seemed that the expression of PD-L1 (one of the ligands of PD-1) was lower in the mUM group [126]. As activated tumor-reactive CD8+ T cells express PD-1, this may suggest that there either is a lack of tumor-antigen specific tumor infiltrating lymphocytes (TIL) in mUM or that they are locally suppressed by other means [127]. In the absence of a type 1 immune response, there is less interferon-gamma driven PD-L1 expression [128]. As the target for anti-PD1 treatment is not expressed in most mUM patients, this provides another rationale for the lack of efficacy of anti-PD1 treatment.

Preliminary data from an ongoing trial comparing the immune infiltrate of mUM and mCM show that in accordance with the previously mentioned trial, the density of CD3+CD8+, as well as the distance from CD8+ lymphocyte to tumor cell, was similar in both tumor types. However, macrophages were less numerous in mUM compared to mCM at baseline; further classification of these macrophages is still ongoing. Interestingly, the preliminary data also showed that enrichment for T cell and inflammatory gene expression was observed in a mUM patient with exceptional overall survival in contrast to an overall low CD8 and the absence of an immune gene expression profile in a patient with the shortest overall survival [129]. This suggests that some mUM are immunogenic, despite earlier reports on the immune infiltrate in primary UM. This notion is also supported by a recently published phase II clinical trial applying adoptive cell therapy to treat mUM patients. Twenty-one mUM patients were treated with autologous TIL. Of the 20 evaluable patients, seven (35%) achieved objective tumor regression (six partial response, one complete response), including mUM patients who had previously failed on anti-CTLA4 and anti-PD1 treatment. There was a strong correlation between clinical response, the autologous tumor reactivity of the infused TIL, and the number of reactive TIL infused. This clearly shows that despite the lack of an ultraviolet radiation signature, mUM do express antigens that are recognized by the adaptive immune system, suggesting that a lack of T cell activation in mUM is related to local immune suppression. Both biopsies prior and after TIL treatment were obtained from these patients, genomic and proteomic profiling is ongoing and whole exomic sequencing is being performed [130]. Despite the impressive overall response rate for patients with mUM, the durability was relatively short when compared to what has been observed in mCM. Moreover, a second phase II study is necessary, where patients with mUM are recruited with adoptive transfer of TIL to confirm the results in a larger cohort (NCT03467516).

Another potentially interesting cell-based therapy is treatment with chimeric antigen receptor (CAR) T cells. In hematological malignancies two CAR-T cell constructs targeting CD19 have been approved, both in the United States and in the European Union. One of the pilot trials currently recruiting melanoma patients uses c-Met as a target antigen (NCT03060356). As c-Met plays an important role in both CM and UM, this might be a promising treatment strategy for both melanoma subtypes.

## 5. Conclusions

Cutaneous and uveal melanoma both arise from melanocytes. However, they are biologically distinct tumor types. In recent years, many new treatment options have become available for patients with advanced cutaneous melanoma, improving the disease free and overall survival. Unfortunately, most of these new treatment options do not show the same responses in patients with metastatic uveal melanoma. Chemokine receptors, which play a role in both tumor growth and the formation of metastases, have shown to be promising new targets. Based on the pre-clinical work with anti-CXCR4 and anti-IGF-1R, as well as the first clinical results with a MET/VEGF receptor inhibitor, several treatment options are now (further) investigated in the clinic. Multiple trials with both UM and CM patients that are treated with HDAC-inhibitors are also ongoing.

Recent studies indicate that the role of the adaptive immune system in primary versus metastatic UM might be very different. Where immune infiltrate in primary uveal melanoma is correlated with a worse overall survival, this difference was so far not seen in metastatic lesions. However, even when immune cells succeed in infiltrating metastatic UM lesions, these cells do not seem to be activated. Adoptive cell therapy trials in mUM indicate that metastatic UM are immunogenic and able to trigger tumor-reactive T cells; however, potentially, they are locally suppressed, similar to what is seen in primary UM.

As there is not yet a gold standard in the systemic treatment of metastatic UM, early detection and enrolment in clinical trials seems crucial.
